# Improving medical residents’ self-assessment of their diagnostic accuracy: does feedback help?

**DOI:** 10.1007/s10459-021-10080-9

**Published:** 2021-11-05

**Authors:** Josepha Kuhn, Pieter van den Berg, Silvia Mamede, Laura Zwaan, Patrick Bindels, Tamara van Gog

**Affiliations:** 1 Department of General Practice, Erasmus Medical Centre, P.O. Box 2040, 3000 CA Rotterdam, The Netherlands; 2 Institute of Medical Education Research Rotterdam, Erasmus Medical Centre, Rotterdam, The Netherlands; 3 Department of Psychology, Education and Child Studies, Erasmus University Rotterdam, Rotterdam, The Netherlands; 4 Department of Education, Utrecht University, Utrecht, The Netherlands

**Keywords:** Calibration, Self-assessment, Diagnostic error, Instructional design, General practice, Feedback

## Abstract

When physicians do not estimate their diagnostic accuracy correctly, i.e. show inaccurate *diagnostic calibration*, diagnostic errors or overtesting can occur. A previous study showed that physicians’ diagnostic calibration for easy cases improved, after they received feedback on their previous diagnoses. We investigated whether diagnostic calibration would also improve from this feedback when cases were more difficult. Sixty-nine general-practice residents were randomly assigned to one of two conditions. In the feedback condition, they diagnosed a case, rated their confidence in their diagnosis, their invested mental effort, and case complexity, and then were shown the correct diagnosis (feedback). This was repeated for 12 cases. Participants in the control condition did the same without receiving feedback. We analysed calibration in terms of (1) absolute accuracy (absolute difference between diagnostic accuracy and confidence), and (2) bias (confidence minus diagnostic calibration). There was no difference between the conditions in the measurements of calibration (absolute accuracy, *p* = .204; bias, *p* = .176). Post-hoc analyses showed that on correctly diagnosed cases (on which participants are either accurate or underconfident), calibration in the feedback condition was less accurate than in the control condition, *p* = .013. This study shows that feedback on diagnostic performance did not improve physicians’ calibration for more difficult cases. One explanation could be that participants were confronted with their mistakes and thereafter lowered their confidence ratings even if cases were diagnosed correctly. This shows how difficult it is to improve diagnostic calibration, which is important to prevent diagnostic errors or maltreatment.

## Introduction

Physicians do not always estimate their diagnostic performance correctly (Costa Filho et al., 2019; Davis et al., 2006; Friedman et al., 2005; Meyer et al., 2013). This inaccurate *diagnostic calibration* (Meyer et al., 2013), the mismatch between diagnostic accuracy and confidence in that diagnosis, can have harmful effects for the patient. Although diagnostic errors can have many causes, including system-related causes, cognitive errors play a substantial role. For example, a review of diagnostic errors in internal medicine (Graber et al., 2005) has estimated that cognitive factors play a role in around 74% of these cases. On the one hand, being too confident in one’s diagnosis might lead to *premature closure* (which is often found to occur in cases of cognitive errors Berner & Graber, 2008; Graber et al., 2005)), where physicians stop considering alternative diagnoses too early. Overconfidence has also been linked to decreased requests for diagnostic tests (Meyer et al., 2013). Being underconfident (i.e., unnecessarily uncertain) in a correct diagnosis, on the other hand, could lead to unnecessary further testing and lengthen the diagnostic process. Furthermore, the ability to correctly self-asses one’s performance can help to identify potential learning needs (see self-regulated learning (Zimmerman, 2008)). Improving diagnostic calibration, therefore, could not only help to prevent diagnostic errors but could also aid physicians’ lifelong learning and allow them to become better performers (Eva & Regehr, 2005; Meyer & Singh, 2019; Zwaan & Hautz, 2019).

Studies from cognitive psychology have shown, that calibration of self-assessments made after performance (Hacker et al., 2008) can be improved by providing students with feedback on their previous performance (Labuhn et al., 2010; Lipko et al., 2009; Nederhand et al., 2019; Rawson & Dunlosky, 2007). The same may be true for improving calibration in a medical context: A study by Nederhand et al. (2018) showed that feedback on previous diagnostic performance improved future diagnostic calibration for medical experts as well as for medical students. In that study, participants diagnosed three cases and rated their confidence, after which some of them got feedback for the case in the form of performance standards, i.e. the correct diagnosis, and others did not get feedback. Subsequently, all physicians took the same test where they diagnosed three new, unrelated cases and rated their confidence. It was found that physicians who had previously received feedback on their diagnostic performance showed better diagnostic calibration on the test cases. However, in this study, they used relatively easy cases (resulting in high diagnostic accuracy) and it has been found that physicians’ calibration is less accurate for difficult cases than for easy cases (Meyer et al., 2013).

Therefore, improving calibration on difficult cases would be even more important in order to prevent diagnostic errors. In clinical practice, physicians do sometimes get feedback in the form of clinician report cards that show some of their performance measures in comparison to colleagues, e.g. mortality after surgery (Shahian et al., 2001). These cards have been found to help physicians improve some medical outcomes (see for example Kahi et al., 2013), but they do not yet exist for improving the diagnostic process. If feedback on diagnostic accuracy would improve diagnostic calibration, it would be valuable to use diagnostic report cards as well. Furthermore, feedback could possibly help as an educational tool for physicians in training to identify their learning needs and learn to estimate their performance better. Less over- and underconfidence in physicians in training, could potentially prevent future errors in clinical practice. In the current study, we aimed to investigate whether feedback (providing the correct diagnosis) can help to improve diagnostic calibration for residents in general practice (GP), i.e. physicians in training to become specialist, when cases are more difficult. Thus, we wanted to test whether the findings by Nederhand et al. (2018) would also show with different cases and participants in a slightly different design. Residents were asked to diagnose a case, rate their confidence in the diagnosis, and then either got the correct diagnosis for the case or moved on to the next case without feedback. We expected that GP residents who got feedback would show more accurate diagnostic calibration than residents who did not get the feedback. Additionally, we measured perceived mental effort when diagnosing the cases as well as perceived case complexity to check that the cases were not (perceived as) too easy.

## Method

### Participants

Ninety-seven residents in their first year of the three-year general practice training at the department of general practice at the Erasmus Medical Centre, Rotterdam, were invited to participate in this study. Sixty-nine of them accepted the invitation and completed the session (54 female; age *M* = 29.29, *SD* = 2.51). The study took place during the usual educational program and participants did not receive compensation.

### Material

Twelve written cases were used in this study, describing different patients with different medical conditions (Table 1). The cases were prepared and validated by experienced general practitioners, and used in previous studies (Kuhn et al., 2020). The study was programmed in Qualtrics software (version 05.2019). For each condition, we made six versions of the program, which presented the cases in different orders. Participants moved through the program self-paced and could only move forwards. Qualtrics automatically recorded the participants’ answers.

**Table 1:** Overview of the chief symptoms and medical conditions that were described in the 12 cases

Chief symptom	Correct diagnosis
Diarrhoea	Chronic pancreatitis
Shortness of breath	Heart failure
Palpitation	Panic disorder
Turn dizziness	Benign Paroxysmal Position Vertigo
Rash/eczema	Scarlet fever
Lower back pain	Spondylodiscitis
Amenorrhea	Pregnancy
Pain in legs	Spinal canal stenosis
Tremor in hand	Multiple sclerosis
Facial paralysis	Bell's palsy
Rash in the face	Rosacea
Vaginal discharge	Bacterial vaginosis

### Design and procedure

The study was conducted in one session in computer rooms at the Erasmus Medical Centre. First, participants were asked to read the information letter on their desk and give written informed consent. Another sheet of paper provided a URL that led to one of the 12 Qualtrics programs. These papers were distributed throughout the room, so that participants were randomly assigned to either the feedback condition (*n* = 34) or the no-feedback (i.e. control) condition (*n* = 35). In the program, they received all instructions required for their condition together with an example-case to get acquainted with the procedure. After that, they started diagnosing the first of the twelve cases.

#### Feedback condition

Participants were shown a case and were asked to read it until they had arrived at one most likely diagnosis. They moved on to the next page where they had to fill in their diagnosis. On the next three pages, they were asked to rate their confidence in their diagnosis, their mental effort invested in solving the case, and the complexity of the case. Those 3 measures were rated on 9-point-Likert scales ranging from 1 (very, very little) to 9 (very, very much). Mental effort and complexity were both used as indicators of how complex the cases were for participants. On the next page, participants were shown the correct diagnosis for the case together with the diagnosis they themselves had given and were asked to compare both diagnoses. When they confirmed that they had compared them, they were able to move on to the next case until all twelve cases had been diagnosed.

After completing the 12 cases, participants were asked about their demographics and prior experience. They were shown a list of the diseases and chief symptoms/complaints that were used in this study, and were asked to rate their prior experience on a 5 point-Likert scale ranging from 1 (I have never seen a patient with this disease, symptom or complaint) to 5 (I have already seen many patients with this disease, symptom or complaint). Finally, participants were given a written debriefing and thanked for their time and effort.

#### Control condition

Participants in the no-feedback control condition followed the same procedure as those in the feedback condition, except they did not receive the information on the correct diagnosis for the case and the request to compare it with their own diagnosis.

### Analysis

The data were analysed using IBM SPSS Statistics 25 for Windows. For all analyses we used a significance level of *α* = 0.05. As a measure of effect size, *ηp*^*2*^ is provided for the analyses of variances, with 0.01, 0.06, 0.14 corresponding to small, medium and large effects (Cohen, 1988).

#### Prior experience

To analyse potential differences in prior experience between the conditions, we computed the mean prior experience ratings for the symptoms and diagnoses used in this study. On both variables, we conducted an ANOVA with condition (feedback/no feedback) as a between-subjects factor.

#### Calibration

Experienced general practitioners independently rated the diagnostic accuracy of the given diagnoses while blinded for the experimental condition, assigning either 1 (correct), 0.5 (partly correct), or 0 (incorrect) points. Each diagnosis was rated by two general practitioners with an ‘excellent’ interrater reliability, *ICC* = 0.96 (Cicchetti, 1994). Afterwards, they would come together and discuss the diagnoses where they had not given the same score until they reached agreement, so that each diagnosis had only one score. To calculate diagnostic calibration, we transformed the confidence ratings to match the scale of the diagnostic accuracy scores (cf. Nederhand et al., 2018): Confidence scores 1–3 were recoded into 0, 4–6 into 0.5, and 7–9 into 1. This adjustment also took into account that participants are usually reluctant to use extreme response on a Likert scale (i.e. central tendency bias).

We then computed calibration in terms of *absolute accuracy* and *bias* measures by subtracting the diagnostic accuracy scores from the transformed confidence ratings (Griffin et al., 2019). Absolute accuracy is the absolute (i.e., unsigned) difference between the two and ranges from 0 (perfect calibration) to 1 (fully inaccurate). Bias is the signed difference between the two and ranges from + 1 (complete overestimation) to − 1 (complete underestimation) with 0 again meaning perfect calibration. Per participant, we calculated the mean absolute accuracy and bias scores across all 12 cases. On both outcome measures, we performed an ANOVA with condition as a between-subject variable. Also, we performed a t-test on mean bias to see if it significantly differed from 0 (i.e., as zero means correct calibration, this analysis will tell whether there was significant underestimation or overestimation).

#### Post hoc exploratory analyses

In an exploratory analysis we took a closer look at calibration in relation to diagnostic accuracy. For each participant, we computed the mean bias on cases diagnosed incorrectly (diagnostic accuracy = 0; cases *n* = 473) and on cases diagnosed correctly (diagnostic accuracy = 1; cases *n* = 341). This may give more insight into differences in overconfidence and underconfidence between the conditions than averaging over the 12 cases. That is, on incorrectly diagnosed cases, participants will either be accurate or overconfident, whereas on correctly diagnosed cases they will either be accurate or underconfident (so by computing the mean bias across the 12 cases, overconfidence and underconfidence might cancel each other out). Note that these means were based on a different number of cases for each participant, depending on the individual performance. Partly correct cases (diagnostic accuracy = 0.5; cases *n* = 14) were left out of this analysis. We performed separate ANOVAs for correct and incorrect cases, with condition as a between-subjects factor.

## Results

### Prior-experience ratings

Table 2 shows the demographics and mean prior experience ratings. The analyses showed no differences between the conditions on mean prior-experience ratings for the diagnoses, *F* (1, 67) = 0.12, *p* = 0.727 *η*_*p*_^2^ < 0.01, and the symptoms, *F* (1, 67) = 0.05, *p* = 0.831, *η*_*p*_^2^ < 0.01, that were used in the cases of this study.

**Table 2:** Demographics and prior experience ratings

	No-feedback condition	Feedback condition	Total
Sample size	35	34	69
Gender	27 female	27 female	54 female
Age, *mean (SD)*	29.23 (2.31)	29.35 (2.73)	29.29 (2.51)
Prior experience with diagnoses, *mean (SD)*	2.38 (.52)	2.43 (.61)	2.41 (.57)
Prior experience with symptoms, *mean (SD)*	3.21 (.55)	3.24 (.64)	3.22 (.59)

Prior experience was rated on a 5-point Likert-scale ranging from 1 (I have never seen a patient with this condition, symptom, or complaint) to 5 (I have seen many patients with this condition, symptom, or complaint)

### Descriptive statistics

Table 3 shows the means for all outcome measures (diagnostic accuracy, confidence, complexity, absolute accuracy, bias). Mean diagnostic accuracy (*M* = 0.42), and mean confidence (*M* = 5.63), mental effort (*M* = 5.07) and complexity (*M* = 5.52) ratings, were at an intermediate level and showed no ceiling- or floor effects.

**Table 3:** Mean and standard deviation for all outcome measures (diagnostic accuracy, confidence in the diagnosis, mental effort, case complexity, and as measures of calibration: absolute accuracy and bias)

	No-feedback condition (*n* = 35)		Feedback condition (*n* = 34)		Total (*n* = 69)	
	Mean	SD	Mean	SD	Mean	SD
Diagnostic accuracy	0.42	0.14	0.43	0.12	0.42	0.13
Confidence rating	5.82	0.80	5.43	0.79	5.63	0.82
Mental effort rating	5.02	1.04	5.12	0.90	5.07	0.97
Complexity rating	5.64	0.82	5.40	0.89	5.52	0.86
Absolute accuracy	0.42	0.12	0.46	0.09	0.44	0.11
Bias	0.22	0.21	0.15	0.20	0.18	0.21

Diagnostic accuracy was scored as either 0 (incorrect), 0.5 (partially correct) or 1 (correct). Confidence and complexity were rated on a 9-point Likert-scale ranging from 1 (very, very low) to 9 (very, very high). Absolute accuracy ranges from 0 to 1. Bias ranges from − 1 to + 1

### Calibration accuracy and bias

The analysis of calibration on all 12 cases^1^, showed no effect of condition on absolute accuracy, *F* (1, 67) = 1.64, *p* = 0.204, *η*_*p*_^2^ = 0.02 or bias *F* (1, 67) = 1.87, *p* = 0.176, *η*_*p*_^2^ = 0.03. The mean bias in the whole sample (*M* = 0.18) significantly differed from zero, *t* (68) = 7.22, *p* < 0.001, and thus showed that on average, participants were slightly but significantly overconfident.

The exploratory analysis (Table 4) of incorrect cases only, which would indicate the degree of overconfidence, showed no effect of condition, *F* (1, 67) = 0.19, *p* = 0.665, *η*_*p*_^2^ < 0.01. The exploratory analysis of correct cases only, which would indicate the degree of underconfidence, showed a significant effect of condition, *F* (1, 67) = 6.55, *p* = 0.013, *η*_*p*_^2^ = 0.09, with the feedback condition being more underconfident (*M* = − 0.35) than the no-feedback condition (*M* = − 0.25).

**Table 4:** Post hoc analysis of confidence and calibration, split up for the cases that were diagnosed correctly or incorrectly

	No-feedback condition (*n* = 35)		Feedback condition (*n* = 34)		Total (*n* = 69)	
	Mean	SD	Mean	SD	Mean	SD
*Incorrect cases (n* = *473)*						
Confidence rating	5.30	1.07	5.11	.86	5.20	.97
Bias	.54	.19	.52	.16	.53	.18
*Correct cases (n* = *341)*						
Confidence rating	6.49	.80	5.90	1.03	6.20	.96
Bias	− .25	.15	− .35	.19	− .30	.17

The number of correct or incorrect cases on which the means are based differs for each participant, depending on their performance

## Discussion

It is important for physicians to be able to correctly estimate their diagnostic performance, as overconfidence in a wrong diagnosis might result in diagnostic error and underconfidence in a correct diagnosis may lead to overtesting. The aim of the current study was to investigate whether providing feedback (in the form of the correct diagnosis for a case), would improve diagnostic calibration for more difficult clinical cases. Against expectations, feedback did not improve diagnostic calibration when compared to the control condition without feedback. Exploratory analyses even showed that the feedback made participants significantly more underconfident on correctly diagnosed cases than participants in the control condition.

This finding is at odds with a recent study in which the same type of feedback was shown to improve diagnostic calibration on relatively easy cases (Nederhand et al., 2018). However, we had different cases and a different study population. Also, they had a learning phase of three cases, that we did not include, but when we analysed only the last nine cases,^1^ leaving the first three cases to learn from the feedback, the results did not significantly differ from those that we reported. Therefore, there may be two explanations why participants in the feedback condition did not profit from seeing the correct answers for the cases and even became underconfident on correctly diagnosed cases. The first explanation is, that as we used more difficult cases, participants in the feedback group were confronted with their mistakes on some cases, and this may have made them more cautious on subsequent cases, resulting in lower confidence ratings regardless of their actual performance. This fits with an explanation proposed by Raaijmakers et al. (2019), who found, similar to our study, that feedback did not help to improve calibration of future self-assessments.

In the study by Nederhand et al. (2018), in which feedback did improve diagnostic calibration, diagnostic accuracy was very high which suggests that all case were easy. Thus, participants in that study might also simply have adjusted their confidence ratings according to their previous performance and stuck with that rating without considering their actual performance on the present case. Given that they were very likely to give a correct diagnosis, this would lead to higher calibration accuracy. This interpretation also fits with findings from studies in which the difficulty of the cases (Meyer et al., 2013) (or items Schraw et al., 1993) does vary, but the confidence ratings do not seem to change according to case difficulty and are rather constant (Hacker & Bol, 2019).

A second explanation for why participants did not benefit from the feedback is that the type of feedback we used, may not be helpful for residents to learn how to judge their own performance. Previously it has been found that simple right/wrong feedback has only limited benefits for improving learning (Ryan et al., 2020). Giving students more elaborate feedback on their performance, that explains why certain answers are right or wrong and the underlying concepts, is more effective for improving performance on future tests (Ryan et al., 2020). The same may be true for improving future calibration. A review by de Bruin et al. (2017) discusses how physicians (in training) may use *predictive cues* to assess their own performance. In order to judge one’s performance, people implicitly make use of a variety of cues (Koriat, 1997). Predictive cues are those cues which help to accurately predict performance, for example when medical experts slow down in clinical practice, they use this as a cue for their difficulty with a case (Moulton et al., 2007). In order for feedback to improve diagnostic calibration, the feedback would need to help physicians to access those predictive cues. We do not yet know what effective predictive cues are for estimating diagnostic performance for physicians in training (de Bruin et al., 2017). However, it has been suggested that providing detailed criteria to judge one’s performance can help improve calibration accuracy (Dunlosky et al., 2011; Hawthorne et al., 2017). In our study, participants only got feedback on the end result, which is the diagnosis, and not on the diagnostic process. Providing a performance standard on both the diagnostic process and the correct diagnosis, could possibly help to not only increase their clinical competence, but also to identify cues in the diagnostic process that help them estimate their performance. Future studies should investigate what possible predictive cues are for physicians in training and whether more elaborate feedback would improve diagnostic calibration.

Our study provides new insides into the effect of feedback on diagnostic calibration, but it also has some limitations that should be considered when interpreting the results. First, the study was conducted with fictive, written cases and the residents’ performance had no further consequences. The results may have differed in a high-stakes context (Hacker & Bol, 2019), for example in medical practice with real patients, when the task is more important for the residents than it is in an experimental setting. Second, we asked participants to choose only one most likely diagnosis and it could be that, if participants gave an incorrect answer, they had the correct diagnosis in mind as a second or third differential diagnosis. This may also contribute to their tendency to be (slightly) overconfident on average. Third, the way participants had to rate their confidence gives us only limited information on their thought processes and behaviours in clinical practice. Future studies could use different descriptors of confidence, similar to Tweed et al. (2020), by asking participants whether they need more knowledge or information to make a decision, would like to consult a colleague, or feel confident to make a decision on their own. These options may also help to teach physicians in training that seeking help is a valid an valuable option, too (although also in this case, being well-calibrated would help to avoid unnecessary help-seeking). Fourth, we only tested general practice residents and we do not know whether the results apply to physicians with more or less experience or physicians from other disciplines, which may also contribute to the different results as compared to Nederhand et al. (2018). Fifth, our study does not give us any information on the sources of miscalibration in physicians in training. Future research could focus on this topic, as it may help to find ways to improve diagnostic calibration.

While our study focussed only on (improvement of) diagnostic calibration, future studies could include an estimation of the medical implications that would results from incorrect diagnoses or inadequate confidence. For instance, in the study by Tweed et al. (2017) participants were asked to answer multiple-choice questions on medical cases and rate their certainty. The answers were scored for their level of safeness. They found that when participants were confident about their answer, their response was likely to be either correct or a response that was not causing any patient harm. However, when a participant gave an incorrect answer, the response was more likely to be unsafe when the participant was very confident about it, resulting in a potentially harmful situation for the patient. Helping physicians to better estimate their performance would be especially important for these situations.

To conclude, addressing how we can improve diagnostic calibration is crucial in order to avoid errors (Meyer & Singh, 2019; Zwaan & Hautz, 2019), but proves to be a complex endeavour. It seems unlikely from our results that providing only feedback on the correct diagnosis for a case, will help physicians to better estimate their diagnostic performance; in fact, we found it can even make them less confident about correct diagnoses. This does not mean, however, that feedback cannot have an important role as an educational tool or in medical practice. Paired with a more elaborate intervention that provides participants with cues that are predictive of their actual performance and include safety implications/ harm, it might still be a helpful tool for learning from mistakes (Meyer et al., 2021; Omron et al., 2018; Schiff, 2008). Future studies should investigate whether such more elaborate feedback interventions would be more effective to improve diagnostic calibration.

## Footnotes

1 Additionally, we analysed only the last nine cases taken together, to give residents the first three cases to learn from the feedback, as did Nederhand et al. (2018). The results did not differ from the analysis of all 12 cases on absolute accuracy, *F* (1, 67) = 0.90, *p* = 0.348, *η*_*p*_^2^ = 0.01 or bias *F* (1, 67) = 2.40, *p* = 0.126, *η*_*p*_^2^ = 0.04.
